# Studies on removal of petroleum fractions from spent petrochemical catalysts to prepare them for pyrometallurgical recovery of Ni, Mo and V

**DOI:** 10.1038/s41598-025-91247-x

**Published:** 2025-03-14

**Authors:** Piotr Madej, Anna Czech, Krzysztof Pęcak, Andrzej Cybulski

**Affiliations:** Łukasiewicz Research Network– Institute of Non-Ferrous Metals, ul. Sowińskiego 5, 44-100 Gliwice, Poland

**Keywords:** Recovery, Nickel, Catalysts, CO_2_ reduction, Critical metals, Solvent extraction, Process chemistry, Environmental chemistry

## Abstract

The article presents a study on the removal of petroleum fractions from reprocessed and decommissioned petrochemical catalysts used in refineries. Petrochemical catalysts are hazardous waste due to their content of petroleum fractions (organic fraction), while also containing high concentrations of Ni, Mo and V. The process of removing petroleum fractions from catalysts was carried out by solvent extraction using hexane. Its purpose was to prepare the catalysts for subsequent steps involving pyrometallurgical processes leading to the recovery of Ni, Mo, V. At the same time, the extraction process was aimed at reducing CO_2_ emissions during melting by deriving the oil fraction before the pyrometallurgical step. The realized studies showed that the degree of removal of the petroleum fraction from catalysts depends on temperature, catalyst/solvent ratio and extraction time. The study showed that it is possible to remove > 40% by weight of the oil fraction initially contained in the catalysts. The research presented in this article is being carried out as part of the LIDER13/0133/2022 project funded by the National Centre for Research and Development, which aims to develop a complete technology for the recovery of Ni, Mo and V from spent petrochemical catalysts.

## Introduction

The observed economic development and energy transition of European Union member states is resulting in an increasing demand for metallic raw materials, which are mainly used in the electronics, energy and electromobility industries^1^.

The announcement of a new transformation plan for the EU economy, described in the “European Green Deal”^2^, assumes that the EU economy is obliged to achieve climate neutrality by 2050, which means achieving zero net greenhouse gas emissions by that year. This will result in the EU economy having to shift from fossil to renewable energy sources. In addition, in Goal 13 “Climate Action” the UN’s “2030 Agenda for Sustainable Development” assumes that CO_2_ emissions will be reduced by 45% by 2030, compared to emissions in 2010^3^. The consequence of this will be a further increase in demand for strategic metals necessary for the EU’s economic development.

Estimates presented by the EU in a document^2^ show that achieving the above-mentioned targets will result in 60 times increase in demand for lithium, 15 times increase in demand for cobalt and 10 times increase in demand for rare earth elements (REEs) in 2050, compared to current supplies for the entire EU economy. At the same time, experts predict 5 times increase in demand for nickel and lithium and 1.5 times increase in demand for cobalt already in 2030^4,5^. This increase is mainly driven by the demand for lithium-nickel-cobalt-manganese (NMC) and lithium-nickel-cobalt-aluminium (NCA) batteries installed as an energy source in electric cars.

At present, one way for the EU to meet the growing demand for Co, Ni, Li and rare earth elements (REEs) is to recover these metals from secondary raw materials. Sources of metals of high significance to the EU economy can include spent petrochemical catalysts used in petroleum refining processes (HDS)^6,7^.

One of the crude oil purification processes is the hydrodesulphurisation (HDS) process. In the HDS process, crude oil products, intermediates and distillation residues are purified from sulphur present mainly in the form of compounds of the type dibenzothiophene (DBT) and its alkyl derivatives. The process is carried out by direct hydrogenolysis of the C-S bond or by hydrogenation of one of the benzene rings, followed by hydrogenolysis of the C-S bond in the intermediate product in the presence of a catalyst^6^.

Hydrodesulphurisation (HDS) processes use catalysts containing the transition metals Ni, Mo, V and Co, where Co and V are the critical metals (CRMs)^2^. These metals in oxide form are deposited on a ceramic substrate made of alumina (γ-Al2O3) or a mixture of alumina and zeolite^6^. As the catalyst is operated for a longer period of time in the HDS plant, it becomes ‘sulphur poisoned’ and consequently loses its catalytic properties and is taken out of service.

The classification of end-of-life petrochemical catalysts as hazardous waste results in them being subject to rigorous treatment restrictions. This results in these catalysts currently being stockpiled. There is no processing of them in Poland or the EU towards metal recovery and, more disturbingly, they are not currently being considered as a source of metals for the EU.

Taking into account the above-mentioned factors, it seems appropriate to develop a technology for processing catalysts from the petrochemical industry that meets the assumptions of a closed-loop economy with a reduced carbon footprint. Therefore, work has been undertaken towards the development of a complete technology allowing the recovery of Ni, Mo and V from this type of material by:

- initial separation of the petroleum fraction by a solvent extraction process (with separation of the hexane from the petroleum fraction, after the solvent extraction process, to produce an alternative fuel),
- then thermal enrichment of the catalysts after the solvent extraction process (pyrolysis and roasting) for the recovery of molybdenum as oxides in the form of dust,
- and final pyrometallurgical treatment to recover Ni, Mo and V as an alloy of these metals.

**Fig. 1:**
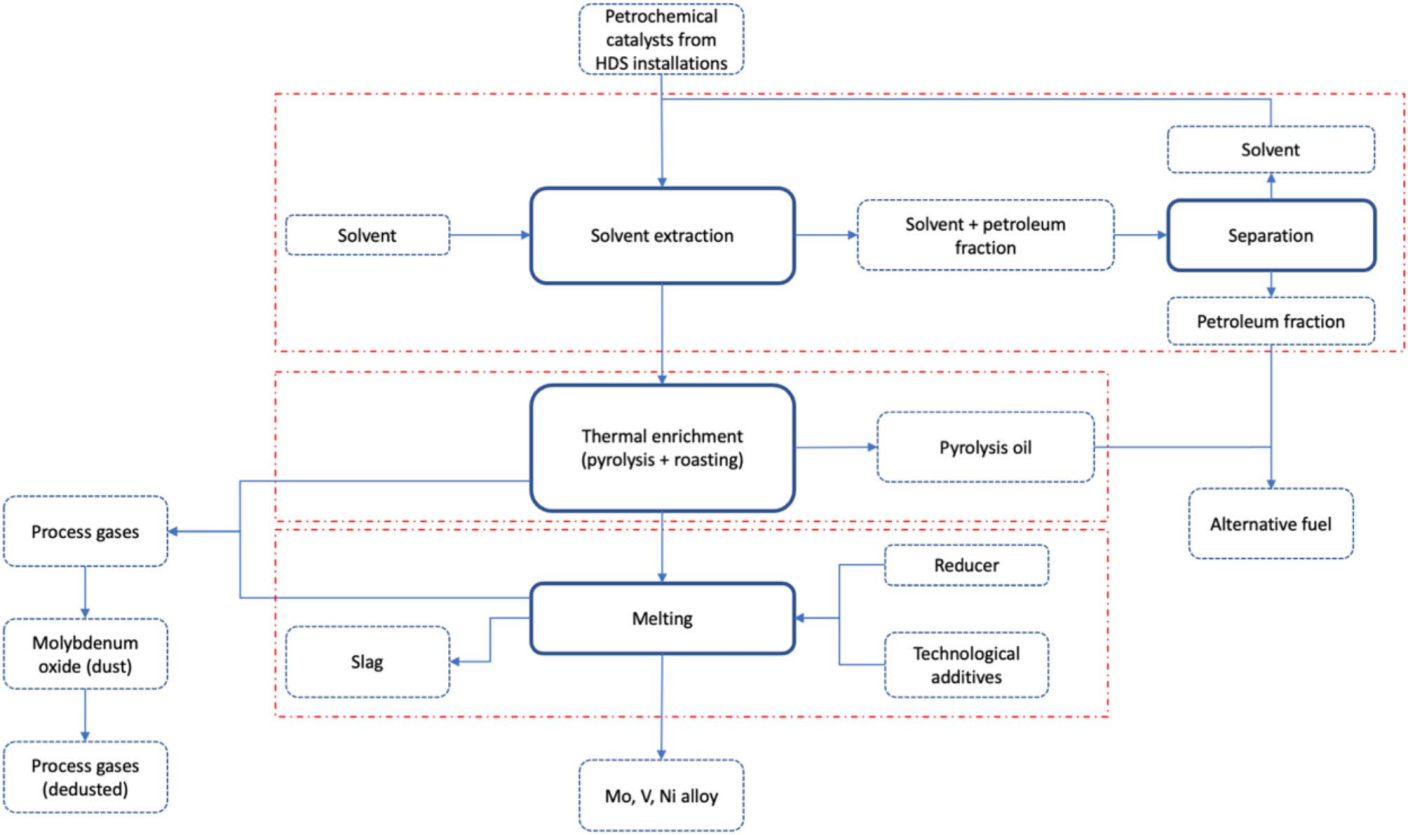
Conceptual technology scheme for the recovery of Ni, Mo and V from end-of-life petrochemical catalysts consisting of three main stages: solvent extraction, thermal enrichment and melting which is being examined as part of the project LIDER13/0133/2022.

A conceptual diagram of the technology is presented in Fig. 1.

This article presents the first part of the research in the technology under development, which aimed to determine the optimum parameters for the solvent extraction process. Solvent extraction is the first stage of the solution under development.

The current demand for critical raw materials essential for the EU’s economic development is mainly covered by imports. The largest supplier of cobalt to the EU is the Democratic Republic of Congo covering 68% of EU demand for this metal. China supplies 78% of the EU’s lithium demand and 55% of the vanadium^2^. This means that the EU economy is currently dependent on imports of strategic raw materials necessary to fulfil the economic transformation goals of the ‘European Green Deal’.

Secondary raw materials of man-made origin can be a source of metals of strategic importance to the EU economy and the implementation of the ‘European Green Deal’. It is therefore important to undertake research into the recovery of Ni, Mo and V from spent petrochemical catalysts used in crude oil processing, as this will allow the European Union to become partially independent of external suppliers, thus increasing Europe’s raw material security.

## Physico-chemical properties of the examined material

The materials for the study were spent, end-of-life petrochemical catalysts from hydrodesulphurisation units with the composition presented in Table 1.

**Table 1:** Comparison of the chemical composition of the material studied with literature data.

Component	Content [wt%]		
	This study	Reference [7]	Reference [6]*
Al	19.02	n.b.	11.59
S	10.07	7.9 ÷ 9.5	2.89
Mo	9.33	4,3 ÷ 11,0	0.59
C	8.69	21.0 ÷ 21.5	54.80
Ni	4.36	2.1 ÷ 8.5	n.b.
V	2.50	1.9 ÷ 17.8	0.38
Fe	0.28	0.1 ÷ 0.3	n.b.
O	n.b.	n.b.	28.88

*Catalyst after removal of the petroleum fraction.

The catalysts came from the gudron hydrodesulphurisation plant (HOG), where vacuum residues from crude oil distillation are processed. The spent, end-of-life petrochemical catalysts granulation was examined nad showed that the catalysts have the 0.5 ÷ 4 mm grain fraction, consisting of irregularly shaped grains from 0.5 to 3 mm and cylinders 1 mm in diameter and 2 ÷ 3 mm in length. The plant consists of two reactors with a so-called embers bed, where gudron containing 3.3% sulphur, 0.65% nitrogen and approximately 215ppm nickel and vanadium is processed^6^.

A further stage was to determine in the form of which compounds the elements were present in the material. For this purpose, the catalysts were subjected to X-ray phase analysis.

**Fig. 2:**
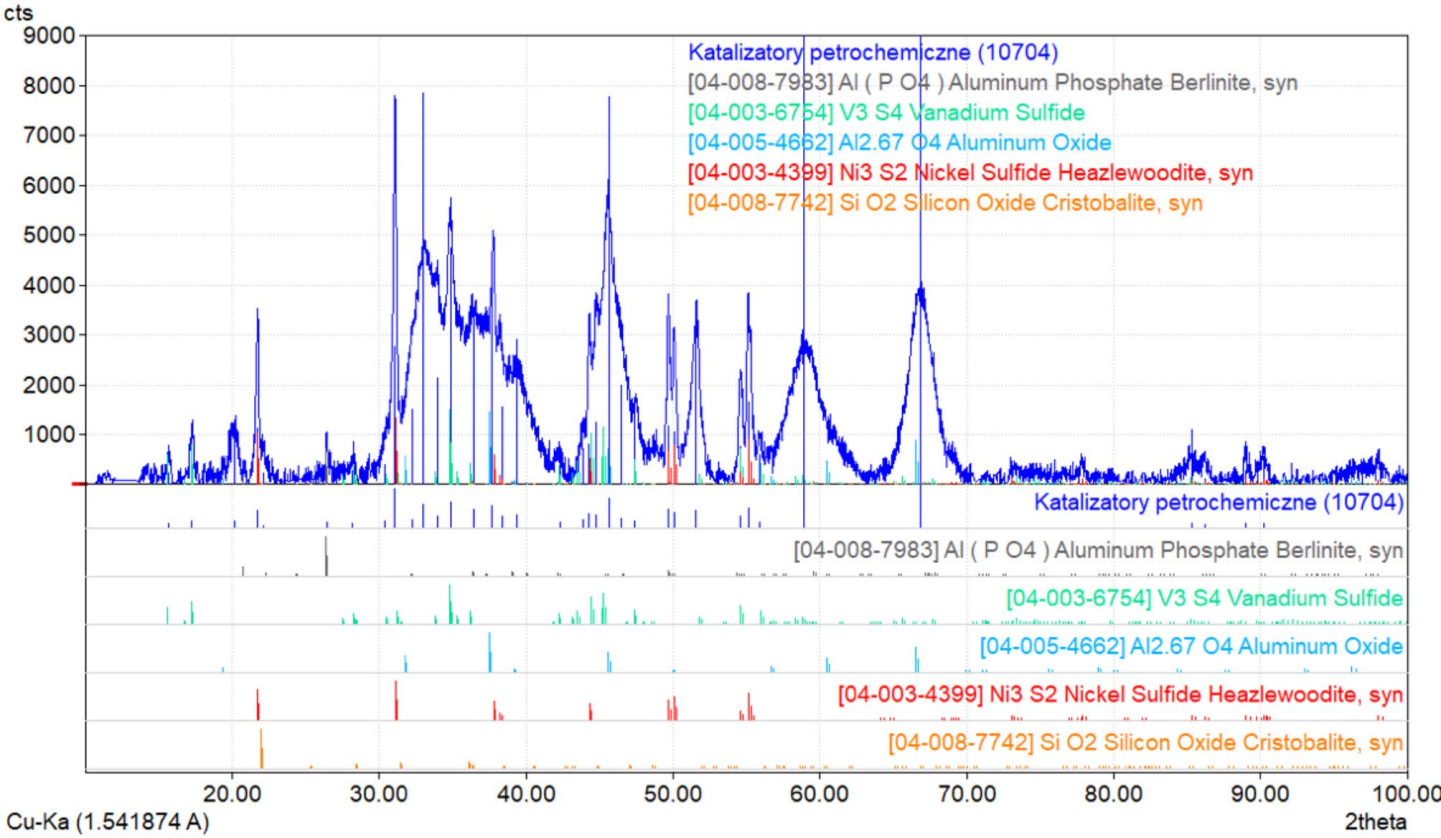
X-ray diffractogram obtained from a sample of spent petrochemical catalyst showing the forms in which each metal occurs (spent petrochemical catalyst; Al(PO_4_); V_3_S_4_; Al_2.67_O_4_; Ni_3_S_2_; SiO_2_).

A qualitative analysis of the phase composition of the samples was performed based on the interpretation of the diffractogram prepared with a Seifert-FPM XRD7 X-ray diffractometer with Seifert software i Match! and ICDD PDF-4 + catalogue data 2023. X-ray characteristic radiation Cu Kα and a Ni filter were used. The analysis was carried out in the 2θ angle range from 10° to 100°. The resulting X-ray diffractometer phase analysis of the test sample is shown in Fig. 2.

The diffractogram obtained indicates the presence in spent petrochemical catalysts of phases such as nickel sulphide Ni_3_S_2_ in the form of heazlewoodite (which has a melting point of 797 °C^8^), vanadium sulphide V_3_S_4_ (which has a decomposition temperature above 750 °C^9–11^), Al_2.67_O_4_, which is a gamma variant of aluminium (III) oxide γ-Al_2_O_3_, aluminium phosphate Al(PO_4_) as berlinint (which has a melting point of about 1800 °C and a decomposition temperature of about 1220 °C^12,13^) and silica SiO_2_ in the form of cristobalite.

**Fig. 3:**
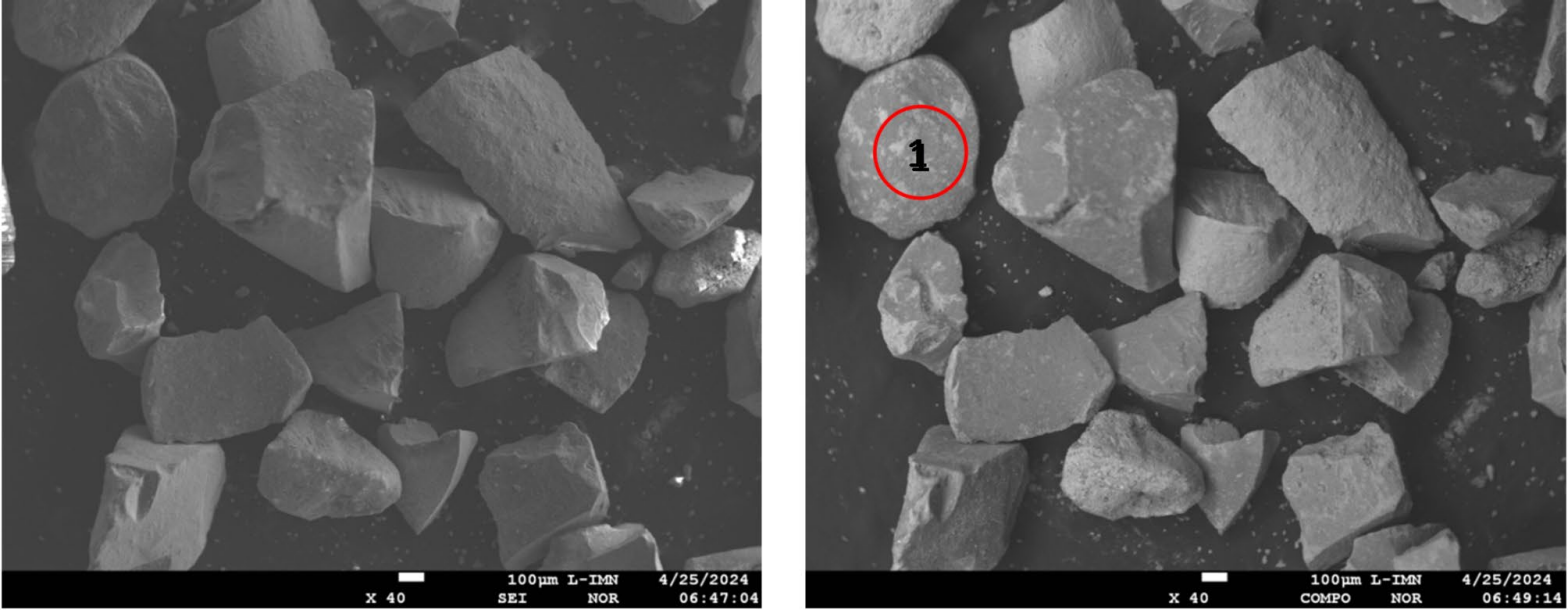
Electron images of petrochemical catalysts taken in SEI mode (contrast depends mainly on surface topography).

In additional, X-ray microanalysis examinations were carried out using an X-ray microanalysis probe on a catalyst sample in the form of a powder applied to a double-sided adhesive carbon slice and by sticking it onto a measuring table. The sample prepared in this way was subjected to tests using a JEOL JXA 8230 X-ray microanalysis probe (15 kV accelerating voltage applied), for which:

- electron imFig. (Fig. 3) in secondary electron light (SEI) and in back-scattered electron light (BSE COMPO),
- elemental distribution maps using the energy dispersive method (EDS),
- quality (energy spectra, EDS) and quantitative analyses of chemical composition (EDS) using standards of all analysed elements for selected areas and points on the sample surface.

**Fig. 4:**
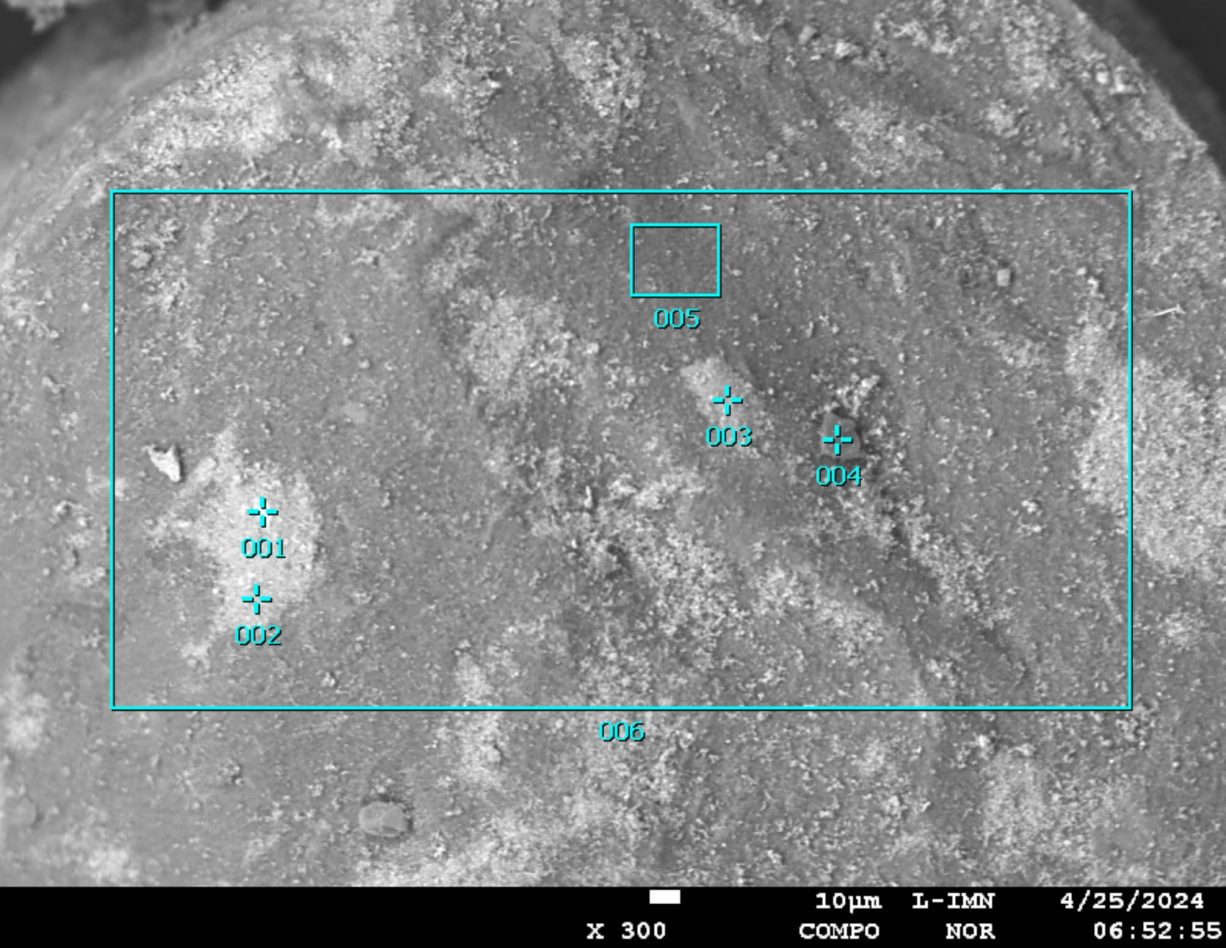
Electron image of the catalyst powder from area 1 of Fig. 3, taken in COMPO mode (reveals differences in chemical composition, the lighter colour corresponding to the heavier element).

**Table 2:** Identified elements in marked points and areas from Fig. 4 (area 1 from Fig. 3).

	Content [%]					
	Point				Area	
	1	2	3	4	5*	6
C	-	-	-	2.5	3.8	6.5
O	-	-	-	29.8	12.5	26.6
Al	0.9	6.0	1.8	28.7	16.7	22.8
P	-	-	-	2.2	1.9	2.4
S	40.8	36.2	36.7	12.0	8.7	15.7
V	40.8	30.7	32.9	6.4	1.5	7.2
Fe	4.7	11.8	11.0	1.1	0.3	1.5
Ni	12.7	10.3	15.3	5.3	3.1	5.4
Mo	-	4.9	1.3	11.9	1.6	12.0
As	-	-	0.4	-	-	-

*Analytical results are standardised to 100%. The area has a very low pass rate (total), which, for example, may indicate the presence of a substance that cannot be analysed by microanalysis (e.g. compounds with hydrogen - organic fraction).

**Fig. 5:**
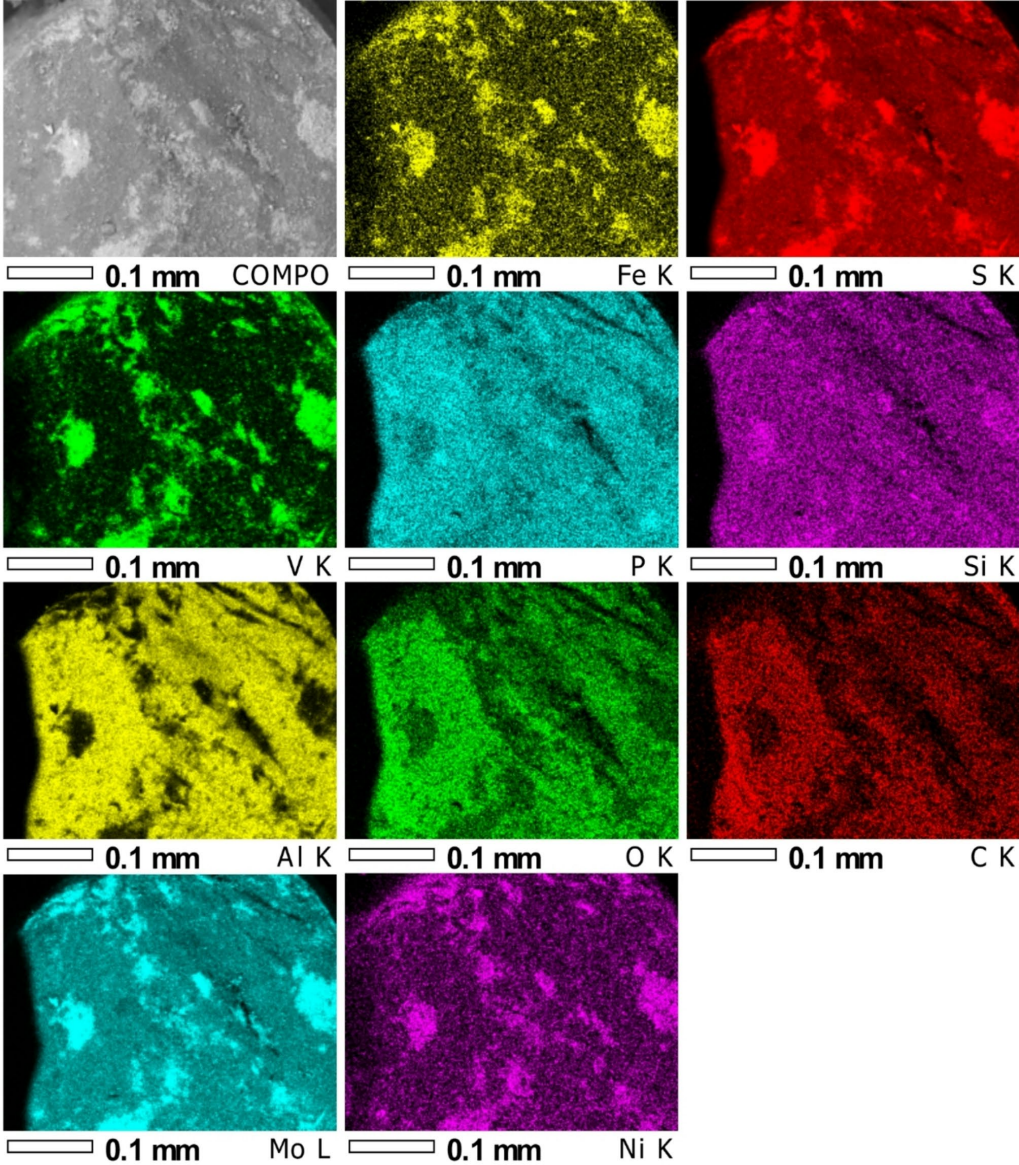
Elemental distribution maps for the surface from the COMPO photo (first left; Fig. 4).

The analyses and element distribution maps performed reveal differences in chemical composition (the lighter colour corresponds to the heavier element). The measurements show that the material has phases consisting of: V + S, Al + O with possible addition of P and S, Si + Mg and S + V + Fe + Ni + Mo.

The point analyses carried out (points from Fig. 4) showed high sulphur and vanadium content, as well as iron, nickel and molybdenum. In contrast, analysis from the surface of two areas showed mainly oxygen, aluminium and sulphur (Table 2). The elemental distribution maps (Fig. 5) confirm this. It can also be seen from the distribution maps that silica and phosphorus are almost homogeneously distributed over the entire area analysed, while aluminium, oxygen and carbon were identified mainly in the dark area of the sample. Analysis of the bright area (Fig. 4) confirms the high concentration of sulphur and vanadium with nickel and iron in these areas.

**Fig. 6:**
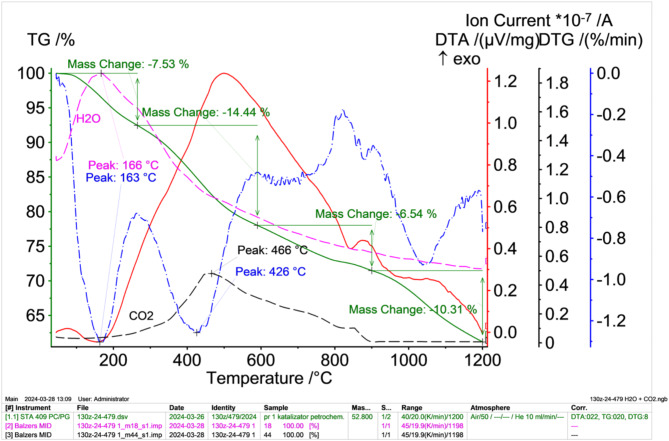
TG (green solid lines), DTA (red solid lines) i DTG (blue dashed dotted line) results of petrochemical catalysts in an oxidising atmosphere after solvent extraction with correlated water (pink dashed dotted lines) and carbon (VI) oxide (VI) (black dashed dotted lines) release intensities obtained from the mass spectrometer.

**Fig. 7:**
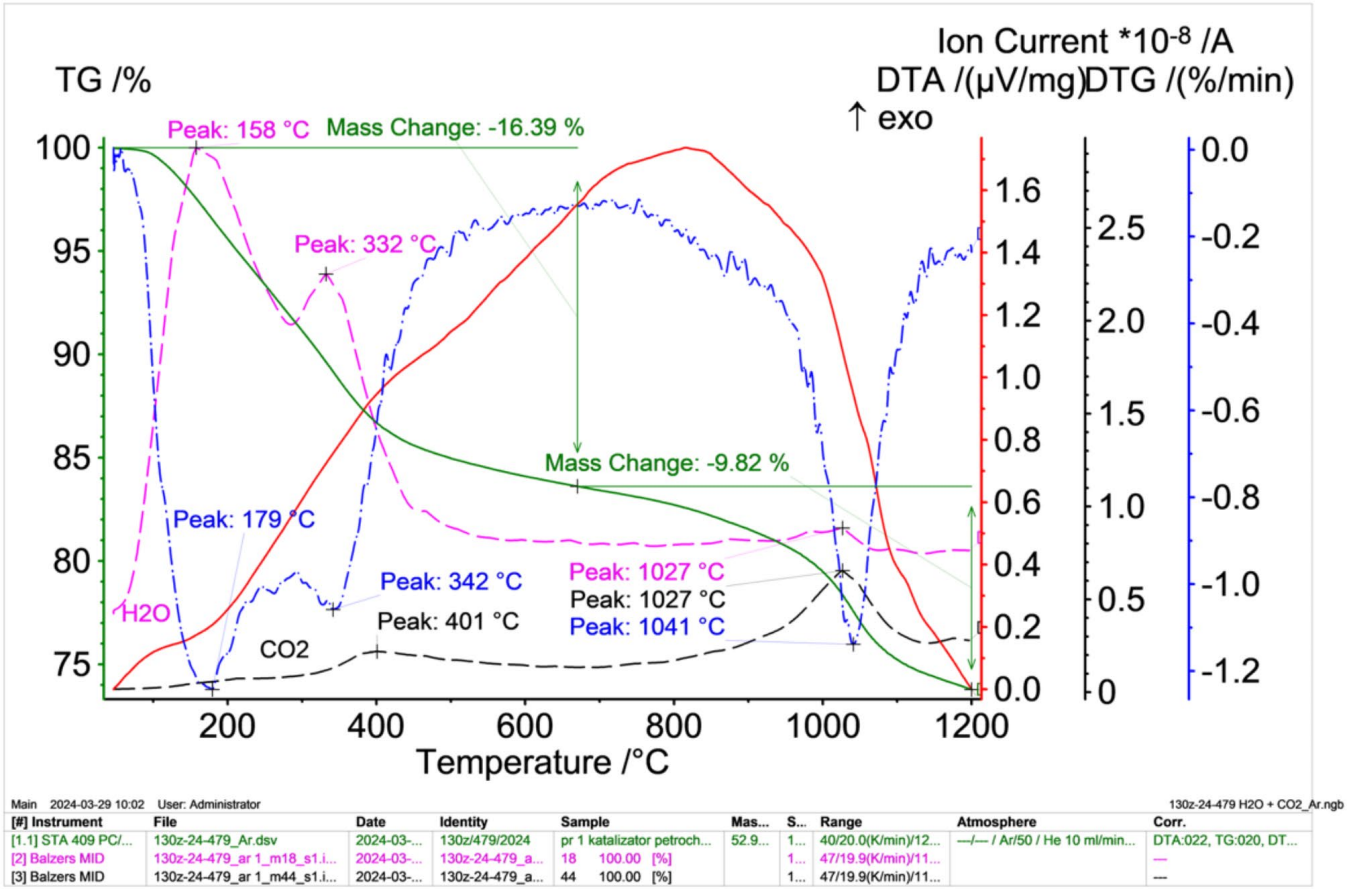
TG (green solid lines), DTA (red solid lines) i DTG (blue dashed dotted lines) results of petrochemical catalysts in an inert atmosphere after solvent extraction with correlated water (pink dashed dotted lines) and carbon monoxide (VI) release intensities (black dashed dotted lines) obtained from the mass spectrometer.

As part of the study to determine the physical and chemical properties of spent petrochemical catalysts, thermogravimetric studies were also carried out, the results of which are presented in Fig. 6 (in an oxidising atmosphere) and Fig. 7 (in an inert atmosphere, argon).

The results obtained from the thermal differential analysis performed in an oxidising atmosphere (Fig. 6) show that, as the sample is heated, there is a decrease in mass, which is initially related to the evaporation of moisture and then to the oxidation (burning) of the petroleum fraction (hydrocarbons) remaining in the catalysts after the solvent extraction process (moisture and the petroleum fraction contained in the capillary pores impossible to remove by drying and solvent extraction). The DTA curve shows an exothermic peak at around 500 °C. This peak may be related to oxidation (combustion) reactions of the petroleum fraction that is released during sample heating. This is evidenced by an increase in the concentration of CO_2_ in the gases, the maximum of which is reached at 466 °C. In this temperature range, a rapid decrease in the mass of the sample is also observed (DTG curve), the maximum of which is reached at 426 °C.

A significant sample weight loss of 14.4% occurs in the temperature range 280–600 °C, which may be related to the significant burning of the petroleum components and the release of sulphur in the form of SO2 (the decomposition temperature of vanadium sulphide V_3_S_4_ is 750 °C and that of nickel sulphide Ni_3_S_2_ is 787 °C). The total mass loss at 1200 °C was 20,649 mg, representing 38.82% of the initial sample mass.

In contrast, the results of thermal differential analysis taken under inert conditions (Fig. 7), show a lower sample weight loss of 26.21%. This is most likely due to slower evaporation of moisture as well as evaporation of volatile components in the form of hydrocarbons and the release of sulphur. The observed exothermic peak in the DTA curve reaches a maximum at about 830 °C (shifted from about 500 °C compared to measurements in an oxidising atmosphere). Carbon monoxide (IV) evolution observed, which has two maxima between 200 ÷ 450 °C, the other above 1100 °C.

## Research methodology

Solvent extraction tests were conducted using an ultrasonic cleaner (CNC Tech model: FTS 360 6 L) to enhance the removal of the oil fraction. This allowed more efficient removal of the petroleum fraction from the catalysts than with standard mechanical stirrers. The reason for this is that the ultrasonic cleaning process uses the phenomenon of cavitation, a process of creating microscopic air bubbles that hit the object being cleaned with high energy, thus breaking up the contaminants (‘petroleum fraction’) on its surface. Hexane was used as the solvent (99% pure for chemical analysis, supplied by Merck Poland). Hexane is a non-polar solvent used in chemical analytics for the determination of hydrocarbons content in process gases (used to leach organic compounds that have been adsorbed on the surface of activated carbon and XAD-2 from process gases).

**Fig. 8:**
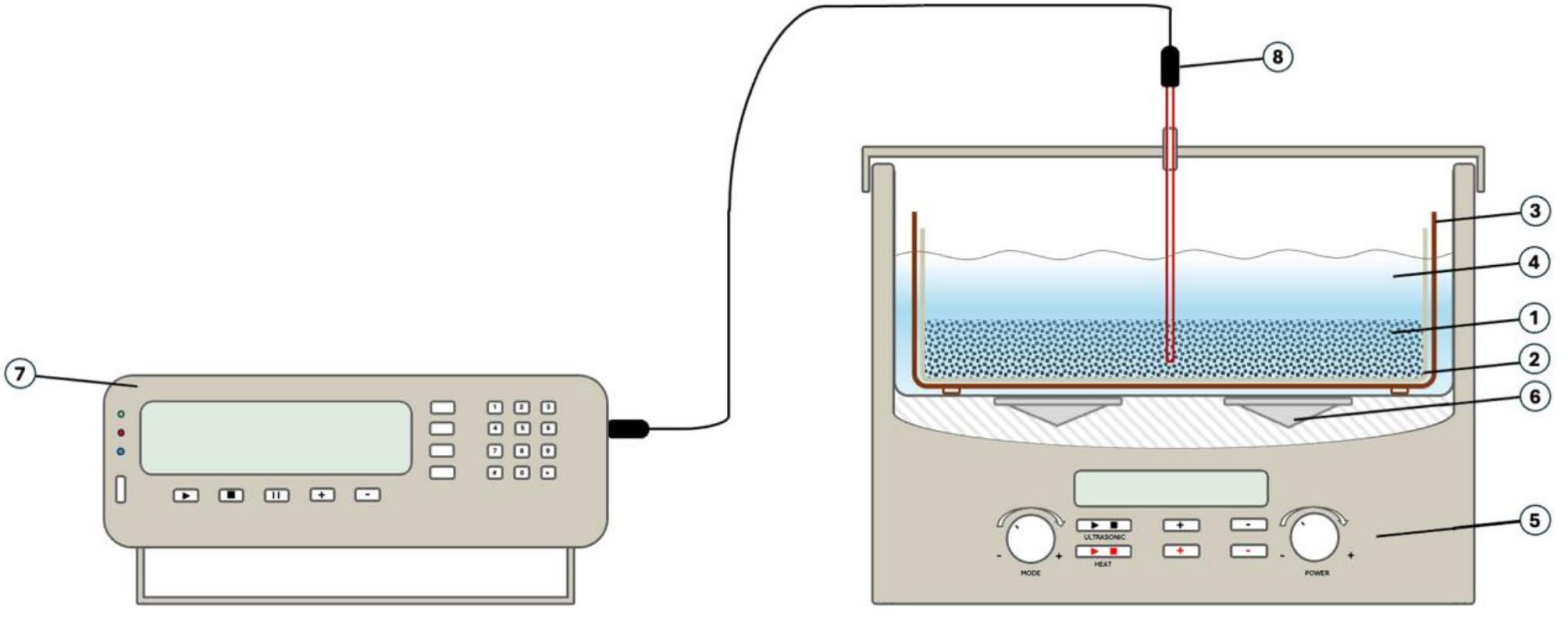
Scheme of test stand for solvent extraction of petroleum fraction from petrochemical catalysts equipped with an multimeter (MPI-CL-16-4) and ultrasonic cleaner.

A schematic view of the test stand for petroleum fraction extraction from spent petrochemical catalysts is presented in Fig. 8.

The main component of the test rig (Fig. 8) for extracting the oil fraction from spent petrochemical catalysts was an ultrasonic cleaner (5) with a working chamber of 6 dm^3^ volume and an ultrasonic frequency of 40 kHz (6) with a control panel enabling control of operating time and temperature. Spent and oily petrochemical catalysts (1) were placed in a thimble made of filter paper (2). The whole was placed in a steel basket (3), which was immersed in solvent (4). Throughout the measurement, the temperature of the solvent was monitored using a K-type thermocouple (NiCr-NiAl, classes 1, +/- 1.5 °C) (8) and recorded every 10 s using a multimeter (METRONIC MPI-CL-16-4) (7).

A 500 g sample of spent catalyst was placed in a thimble and immersed in a solvent (of the appropriate mass for a given Vs/Vl ratio for a particular measurement). The parameters for a particular measurement (frequency − 40 kHz, extraction time and temperature) were then set on the control panel of the ultrasonic cleaner and the measurement was started. The temperature of the solvent was monitored throughout the measurement. The solvent extraction tests consisted of four measurement series:

- SERIES I - studies on the effect of the ratio of the solid fraction (catalyst) Vs to the liquid fraction (solvent) Vl,
- SERIES II - studies of the effect of temperature T of the extraction process on the degree of removal of the petroleum fraction,
- SERIES III - studies on the effect of time t needed to extract the petroleum fraction from the catalyst,
- SERIES IV - tests of the influence of optimal parameters (determined in SERIES I ÷ III) on the degree of removal of the petroleum fraction.

## Results and discussion

Literature data indicate that petrochemical catalysts can contain up to 25 wt% of petroleum fraction^14^. Using this assumption, up to 125 g of petroleum fraction may be present in 500 g of extracted catalyst. Taking this into account, Tables 2, 3 and 4 present the results of the tests carried out, together with the calculated removal rates of the petroleum fraction.

**Table 2:** Removal rate of petroleum fraction contained in spent petrochemical catalysts by solvent extraction as a function of catalyst/solvent weight ratio V_s_/V_l_.

V_s_/V_l_	Mass of catalyst	Mass loss (petroleum fraction removed)	Mass of petroleum fraction in the catalyst (according to^7^)	Petroleum fraction removal rate	Increase in extraction efficiency depending on V_s_/V_l_
	[g]	[g]	[g]	[%]	[%]
t = 5 min.					
1:1	500.278	19.14	125.07	15.3	100
1:2	500.012	21.71	125.00	17.4	113
1:3	500.116	26.58	125.03	21.3	139
1:4	500.154	25.33	125.04	20.3	132
1:5	500.122	26.93	125.03	21.5	141
1:6	500.132	24.61	125.03	19.7	129
t = 20 min.					
1:1	500.278	32.18	125.07	25.7	100 (168)
1:2	500.012	36.37	125.00	29.1	113 (168)
1:3	500.116	41.25	125.03	33.0	128 (155)
1:4	500.154	46.18	125.04	36.9	143 (182)
1:5	500.122	48.41	125.03	38.7	150 (180)
1:6	500.132	47.19	125.03	37.7	146 (192)

( ) – increase in extraction efficiency depending on V_s_/V_l_ calculated with reference to t = 5 min.

Measurements of the effect of the catalyst/solvent weight ratio V_s_/V_l_ on the extraction rate of the petroleum fraction showed that the optimum V_s_/V_l_ ratio for which the highest extraction rate is obtained is 1:5. For this ratio, both for t = 5 min and for t = 20 min., the best results were obtained, with 26.93 and 48.41 g of petroleum fraction removed, respectively. The increase in extraction efficiency (calculated in relation to the weight of the oil fraction removed for the measurement at V_s_/V_l_ =1:1) for this ratio was 141% and 150%, respectively. The measurement results also clearly show that an increase in extraction time increases the petroleum fraction removed. For individual V_s_/V_l_ ratios, increasing the extraction time from 5 to 20 min. resulted in an increase of 155 ÷ 192% in the degree of petroleum fraction removed.

**Table 3:** Removal rate of petroleum fraction contained in spent petrochemical catalysts by solvent extraction for the study of the effect of temperature T on the process (V_s_/V_l_=1:5).

Temperature	Mass of catalyst	Mass loss ( petroleum fraction removed)	Mass of petroleum fraction in the catalyst (according to [7])	Petroleum fraction removal rate	Increase in extraction efficiency depending on T
[°C]	[g]	[g]	[g]	[%]	[%]
t = 5 min.					
25	500.048	23.26	125.01	18.6	100
30	500.076	29.48	125.02	23.6	126
40	500.054	27.78	125.01	22.2	119
50	500.046	28.92	125.01	23.1	124
60	500.072	32.77	125.02	26.2	141
t = 20 min.					
25	500.048	42.26	125.01	33.8	100 (181)
30	500.034	43.42	125.01	34.7	102 (147)
40	500.042	43.07	125.01	34.5	101 (155)
50	500.026	46.43	125.01	37.1	109 (160)
60	500.028	46.13	125.01	36.9	109 (141)

( ) – T-dependent increase in extraction efficiency calculated with reference to t = 5 min.

The results in Table 3 show that an increase in process temperature promotes extraction efficiency. For t = 5 min, an increase in temperature from 25 °C to 60 °C increased the extraction efficiency to 141%. In contrast, for t = 20 min, the increase was up to 109%. The best extraction results were obtained for t = 5 min and temperature T = 60 °C, while for t = 20 min and temperature T = 50 °C. However, it should be noted that the difference in extraction efficiency for t = 20 min. at 50 °C and 60 °C is small at 0.7%.

**Fig. 9:**
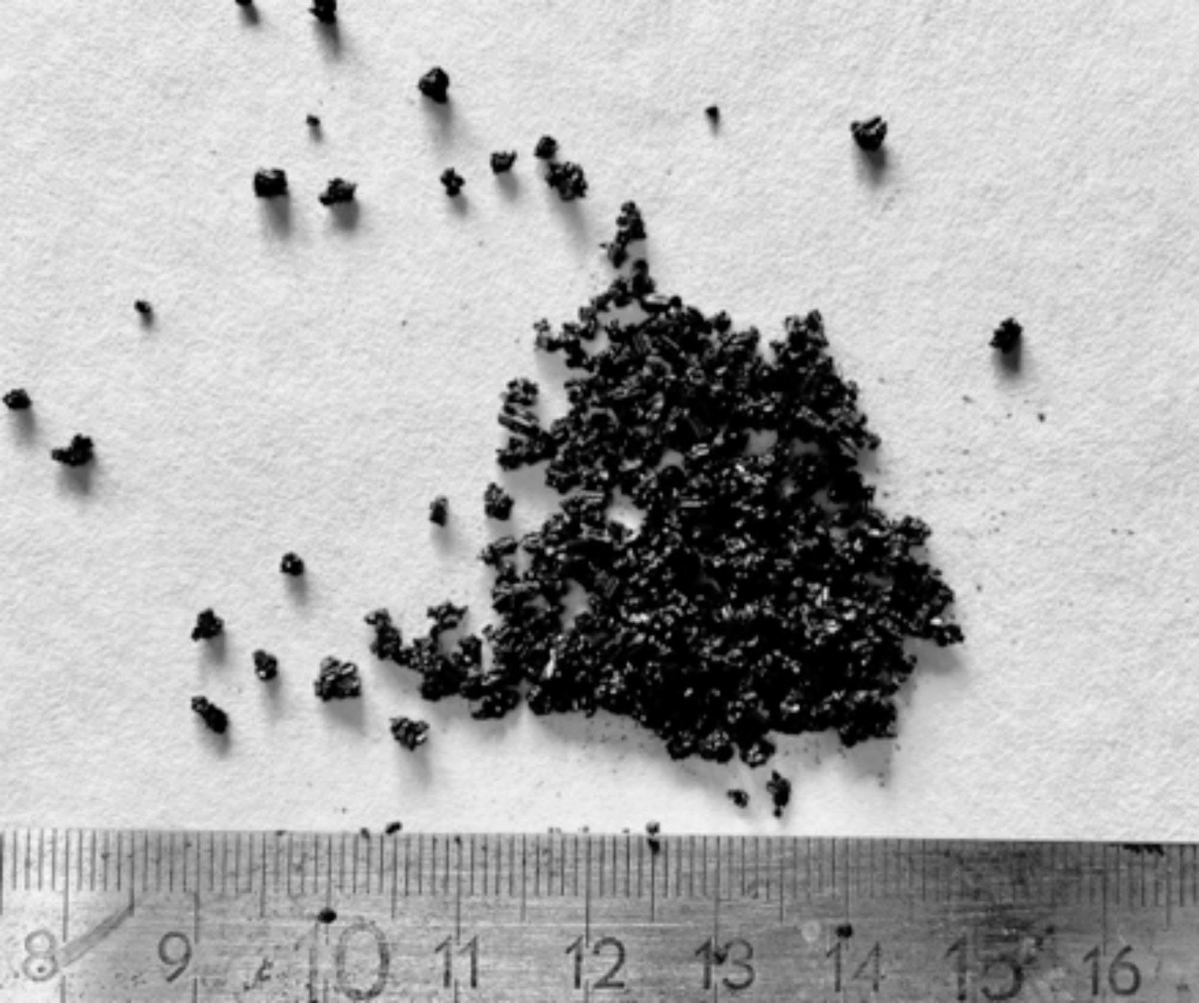
‘Raw’ petrochemical catalysts before the extraction process.

**Fig. 10:**
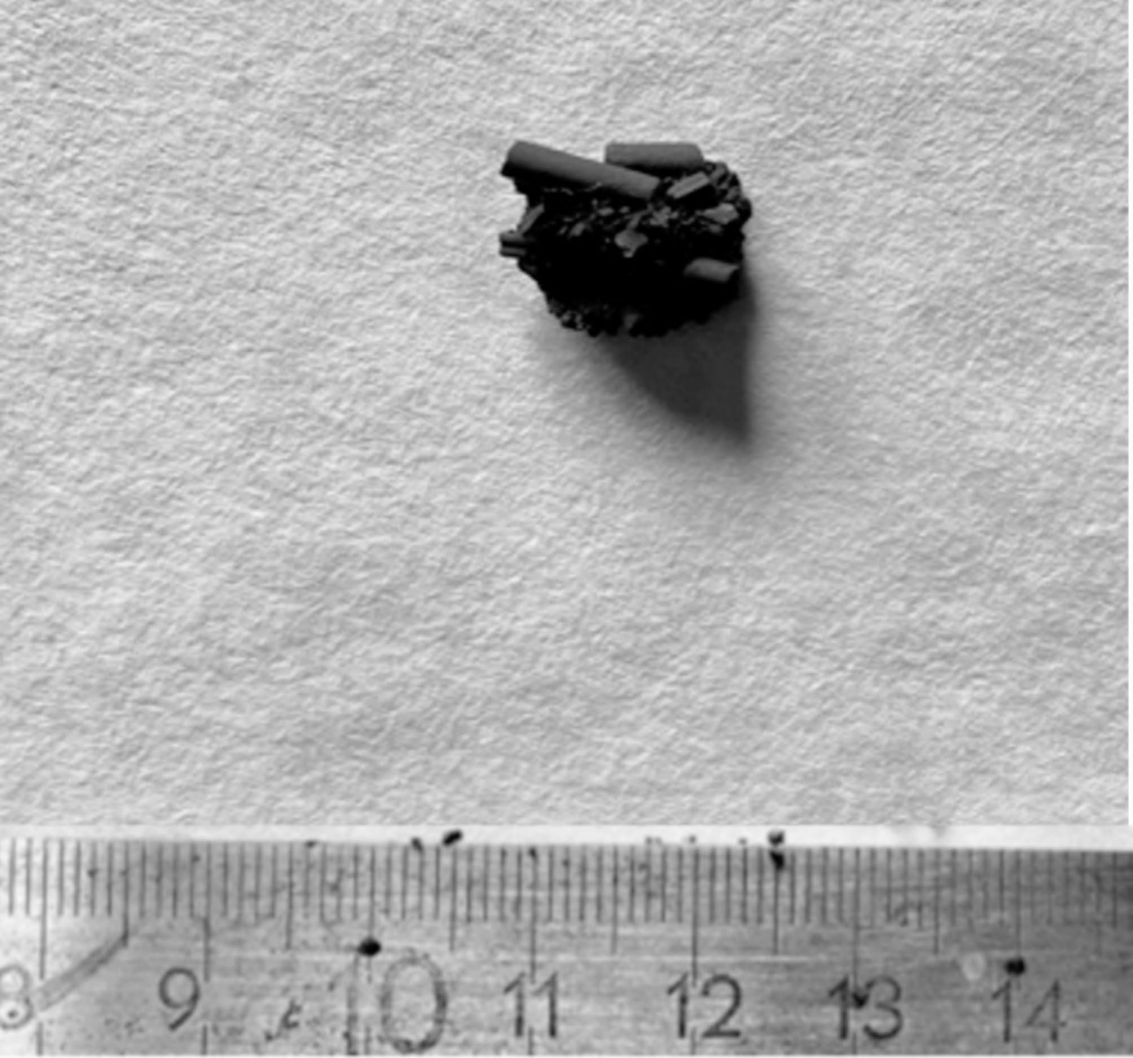
Granule (conglomerate) after the extraction process.

An explanation for this phenomenon is that, after measurement, granules in the form of conglomerates from single catalysts were observed in the sample of catalysts (Fig. 9), which do not disintegrate during the extraction process (Fig. 10) due to the ultrasonic effect. This causes the solvent to be unable to enter the granule and remove the petroleum fraction, which affects the total mass of hydrocarbons removed during the solvent extraction process for a given measurement.

The mass of the granule shown in Fig. 10 was approximately 1.5 g (weight measurement of granule by AXIS ACZ6200 laboratory balance; weight measurement accuracy +/- 0.01 g). Assuming that about 25% of its mass is the petroleum fraction, this gives 0.375 g of hydrocarbons contained in the granule. Considering that the difference in mass removed between T = 50 °C and 60 °C for t = 20 min is 0.296 g. It can therefore be concluded that the difference between the mass of the petroleum fraction removed at these temperatures for t = 20 min is due to the presence of granules in the catalyst sample that was tested at T = 60 °C. For T = 50 °C and for t = 5 min, no granules were observed to interfere with the results obtained.

Increasing the extraction time from t = 5 min. to t = 20 min. increased the amount of petroleum fraction removed from 141 to 181% (calculated in relation to the mass of petroleum fraction removed for measurements at t = 5 min.).

**Table 4:** Removal rate of the petroleum fraction contained in spent petrochemical catalysts by solvent extraction for the study of the effect of time t on the process (V_s_/V_l_=1:5; T = 60 °C).

Time	Mass of catalyst	Mass loss (petroleum fraction removed)	Mass of petroleum fraction in the catalyst (according to [7])	Petroleum fraction removal rate	Increase in extraction efficiency depending on t
[min.]	[g]	[g]	[g]	[%]	[%]
5	500.072	32.77	125.02	26.2	100
10	500.090	40.25	125.02	32.2	123
20	500.038	43.27	125.01	34.6	132
30	500.010	48.88	125.00	39.1	149
40	500.040	53.77	125.01	43.0	164
50	500.062	55.79	125.02	44.6	170
60	500.050	57,34	125.01	45.9	175
90	500.060	57.15	125.02	45.7	174
180	500.290	54.65	125.07	43.7	167

A study of the effect of time on the extraction process shows that increasing the time increases the extraction efficiency of the petroleum fraction from the catalysts. The removal rate of the petroleum fraction for T = 60°C and Vs/Vl = 1:5 increases with increasing extraction time from 26.2% at 5 min to 45.9% at 60 min. Increasing the extraction time to 180 min did not result in a further increase in the removal of the petroleum fraction, which means that the optimal extraction process time is 60 min. This is explained by the fact that the remaining petroleum fraction is contained in the capillary pores on the surface of the catalyst, where the hexane is unable to reach and thus the remaining organic fraction cannot be removed. The maximum increase in extraction efficiency was obtained for time t = 60 min and was 175%. A further increase in extraction time did not result in an increase in extraction rate (174% and 167%).

Most research work on the recovery of metals from spent petrochemical catalysts has focused on their processing by pyrometallurgical methods through remelting to obtain Fe-Ni-Mo-V alloy^7,14,15^. Studies on the removal of the petroleum fraction by solvent extraction were carried out in^6^, where dichloromethane, anthracene oil, benzene, tetrahydrofuran, toluene and tetrahydrofuran were used. The best extraction results were obtained for dichloromethane where 62.4% of the petroleum fraction was removed. This is 35.9% more of the petroleum fraction removed than in the study presented in this article, but the sample of catalyst used in the paper^6^ being extracted was only 20 g, and the process itself was carried out in a Soxhlet apparatus at the boiling point of the solvent in use.

## Conclusions

The solvent extraction research carried out has clearly shown that it is possible to remove the petroleum fraction from spent, end-of life petrochemical catalysts using hexane. The optimum process parameters for which the removal rate of the petroleum fraction is highest are catalyst/solvent mass ratio V_s_/V_l_=1:5, process time t = 60 min and temperature T = 60 °C. Five additional tests were carried out for the determined optimum conditions, which showed that between 43.9% and 44.7% of the mass of the petroleum fraction initially contained in the catalysts was removed.

The proposed petroleum fraction removal method allows at least 40% of the initial mass of the petroleum fraction to be removed from the petrochemical catalysts. The chemical analysis carried out shows that there is 8.69% carbon in the material before the process (Table 1). This results in solvent extraction reducing CO_2_ emissions (during melting of this material) by 127.4 kg for each Mg of catalysts directed to the pyrometallurgical process, by removing carbon (in the form of the petroleum fraction) at an earlier stage.

## Data Availability

The datasets generated and/or analysed during the current study are not publicly available as these data belong to the Lukasiewicz Research Network - Institute of Non-ferrous Metals, but are available from the author responding to a reasonable request after obtaining permission from the management of the Lukasiewicz Research Network - Institute of Non-ferrous Metals.Requests for data sharing should be addressed to Mr. Grzegorz Krawiec at: grzegorz.krawiec@imn.lukasiewicz.gov.pl.
